# A Case of Enterococcal Patent Ductus Arteriosus-associated Endarteritis in a Preterm Neonate

**DOI:** 10.1097/INF.0000000000004232

**Published:** 2024-01-18

**Authors:** Alessandra Mayer, Beatrice Letizia Crippa, Carlo Pietrasanta, Irene Picciolli, Andrea Ronchi, Roberto Raschetti, Alessandra Bandera, Anna Maria Colli, Fabio Mosca, Gaia Francescato, Lorenza Pugni

**Affiliations:** * From the NICU, Mother and Child Department, Fondazione IRCCS Ca’ Granda Ospedale Maggiore Policlinico; † Department of Clinical Sciences and Community Health, University of Milan; ‡ Medical Department, Infectious Diseases Unit, Fondazione IRCCS Ca’ Granda Ospedale Maggiore Policlinico; § Department of Pathophysiology and Transplantation, University of Milan; ¶ Cardio-Thoraco-Vascular Department, Cardiology Unit, Paediatric Cardiology Service, Fondazione IRCCS Ca’ Granda Ospedale Maggiore Policlinico, Milan, Italy.

**Keywords:** endocarditis, endarteritis, patent ductus arteriosus, enterococcus, neonate

## Abstract

**Introduction:**

The persistent patency of the ductus arteriosus frequently occurs in premature neonates and can cause infective endocarditis (IE) or ductal endarteritis (DE) during sepsis. Even though neonatal IE and DE are believed to be a rare eventuality, their incidence has been increasing in the last decades due to the improved survival of even more preterm babies, favored by highly invasive procedures and therapies. In parallel, antimicrobial resistance is another rising problem in neonatal intensive care units, which frequently compels to treat infections with broad-spectrum or last generation antibiotics.

**Case Presentation:**

We report the case of a preterm neonate affected by patent ductus arteriosus-associated DE that followed an episode of sepsis caused by a high-level aminoglycoside-resistant enterococcus. The neonate was successfully treated with the synergistic combination of ampicillin and cefotaxime.

**Discussion:**

IE and patent ductus arteriosus-associated DE are rising inside neonatal intensive care units and neonatologists should be aware of these conditions. Enterococcal IE and patent ductus arteriosus-associated DE sustained by high-level aminoglycoside-resistant strains can be successfully treated with the synergistic combination of ampicillin and cefotaxime even in preterm neonates.

The persistent patency of the ductus arteriosus frequently occurs in premature neonates and can cause, in addition to other adverse events, infective endocarditis (IE) or ductal endarteritis (DE) during sepsis. IE rarely occurs in neonates, although its true incidence in the neonatal population is difficult to estimate since the literature on this topic is limited to dated single case reports or case series.^1,2^ In a relatively recent pediatric dataset, IE diagnosed in the first month of life accounts for 7% of all pediatric cases.^3^ Nevertheless, the incidence of neonatal IE has increased in recent years.^4^ The improved survival of severe preterm infants with central venous catheters in place, beyond the improvement of echocardiographic diagnostic techniques, may explain that.^5^ In fact, most cardiac lesions usually involve the right side of the heart, supporting the hypothesis that central venous catheters represent the major risk factor for neonatal IE, due to mechanical trauma leading to endocardial or valvular endothelial injury. Even the persistent patency of the ductus arteriosus in premature infants can favor the onset of endocarditis,^6^ as occurs with congenital heart defects, but can also cause DE. To our knowledge, most cases of patent ductus arteriosus-associated ductal endarteritis (PDA-DE) are reported in older children and adults, while a single report on 2 neonatal cases has been published.^6^

Pathogens most often involved in neonatal IE and PDA-DE are *Staphylococcus aureus*, coagulase-negative staphylococci and *Candida* species; enterococci, streptococci and Gram-negative organisms have been more rarely reported as causative agents.^7–10^ High-level aminoglycoside-resistant (HLAR) enterococci are increasingly prevalent in the hospital setting and preclude the use of the synergistic combination of a β-lactam and an aminoglycoside, posing a therapeutic challenge especially in neonatal infection.^11^

Here, we report the case of a HLAR enterococcal PDA-DE affecting a preterm neonate.

## CASE PRESENTATION

**FIGURE 1.**
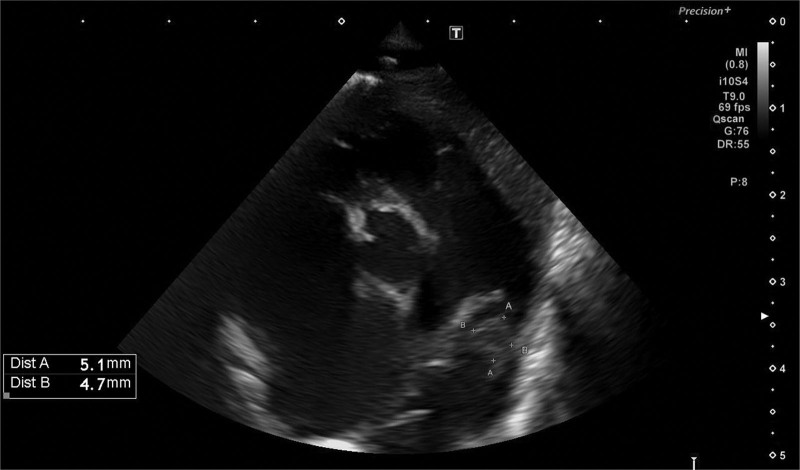
Echocardiogram (ductal view) showing the vegetation inside the ductus arteriosus.

**FIGURE 2.**
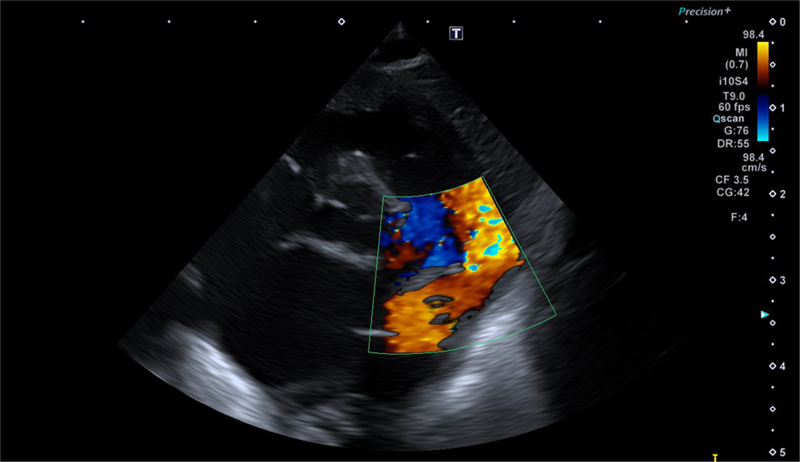
Echocardiogram (ductal view, color Doppler) showing partial obstruction of the ductus arteriosus due to the vegetation.

We report the case of a preterm male neonate, born at 30 weeks of gestational age by urgent cesarean section due to twin anemia-polycythemia sequence in monochorionic twin pregnancy. Delivery room resuscitation with positive pressure ventilation was required for stabilization. Apgar scores were 8 and 8 at 1 and 5 minutes, respectively. Birth weight was 1150 g (8th percentile). After stabilization, the infant was admitted to neonatal intensive care unit due to prematurity and respiratory distress syndrome requiring early surfactant administration and support ventilation (continuous positive airway pressure). Venous umbilical catheter was inserted shortly after admission to neonatal intensive care unit and removed on day of life (DOL) 4, when a central venous line was peripherally inserted. Due to worsening hypercarbia and hypoxemia, the respiratory support was later switched to noninvasive ventilation with neutrally adjusted ventilation assist. Inhaled nitric oxide was required to treat pulmonary hypertension from DOL 4 to 8. After resolution of pulmonary hypertension, echocardiographic signs of hemodynamic significance of the ductus arteriosus appeared. First, ibuprofen (4 doses, from DOL 10 to 13) and then indomethacin (3 doses, from DOL 15 to 16) were administered, without any therapeutic success. Diuretic therapy and fluid restriction were then undertaken, and the patient was scheduled for percutaneous ductal closure. The procedure had to be postponed due to incidental finding on preoperative blood examination of increased C-reactive protein levels (5.19 mg/dL, normal values <0.5 mg/dL), in the absence of clear signs of clinical deterioration. The white blood cell count was 24.48 × 10^9^/L and platelet count was 115 × 10^9^/L. The patient was started on empiric broad-spectrum antibiotic therapy for neonatal late-onset sepsis with amikacin and vancomycin (DOL 28). As blood culture grew ampicillin-sensitive *Enterococcus faecalis*, amikacin and vancomycin were replaced by ampicillin (80 mg/kg/dose every 6 hours) according to sensitivities (DOL 30). A routine echocardiography to monitor hemodynamic impact of PDA during sepsis was then performed and a 4 × 5 mm hyperechogenic image suspicious for thrombus was incidentally found inside the ductus (Figs. 1–2). The patient was therefore started on low-molecular-weight heparin. Nevertheless, the persistency of 2 more positive blood cultures despite targeted antibiotic therapy and central venous catheter removal made the finding more consistent with endarterial vegetation secondary to *E. faecalis* bacteremia. Cefotaxime (50 mg/kg/dose every 8 hours) was therefore added to antibiotic therapy, considering the antibiogram showing high-level gentamicin resistance (DOL 36). Blood culture on DOL 45 was negative, while C-reactive protein and platelet count progressively had normalized as well. Serial echocardiography showed progressive slow decrease of the vegetation size. On DOL 66, after 1 month of antibiotic therapy, no residual ductal shunt could be detected. Heparin was stopped. A 6-week course of combined antibiotic therapy was completed anyway. No cerebral septic embolization was documented at brain magnetic resonance imaging. The patient developed chronic lung disease of prematurity and was transferred to a hospital closer to his home on humidified high-flow nasal cannula at a corrected age of 1 month.

## DISCUSSION

Both IE and DE are rarely diagnosed in neonates and their recognition can be a true challenge for neonatologists.

IE is believed to originate from an injury of endocardium or valvular endothelium, which causes local formation of a sterile thrombus. This condition is called nonbacterial thrombotic endocarditis.^2^ Sick preterm infants are particularly prone to thrombus formation due to the combination of a high prothrombotic activity, low levels of natural anticoagulants and various imbalances in the fibrinolytic systems.^12^ Hemodynamic factors can also contribute to nonbacterial thrombotic endocarditis pathogenesis: blood turbulence, which can be caused, for example, by valvular stenosis or small septal defect, can favor thrombus formation.^2^ Nonbacterial thrombotic endocarditis represents the predisposing condition for IE: indeed, thrombi may be colonized by bacteria or fungi and become infected, resulting in IE.^2^ PDA-DE recognizes the same pathogenetic mechanism. PDA-DE usually affects the pulmonary end of the ductus arteriosus, where blood turbulence and Venturi effect occur.^6^ However, only 2 neonatal cases of PDA-DE were reported to date.

Diagnosis of neonatal IE and PDA-DE can be extremely challenging, since clinical presentation may be subtle and indistinguishable from neonatal sepsis, as in the case described. They should be suspected in case of persistent positive blood culture despite targeted antibiotic therapy, persistent thrombocytopenia or new onset murmur. Microscopic hematuria was also reported in some neonatal cases.^7^

In this scenario, echocardiography is essential for diagnosis. While transesophageal echocardiography is more sensitive than transthoracic echocardiography in detecting endocarditis in adults, transthoracic echocardiography is usually sufficient for detection of endocarditis in children.^13^ However, it might be difficult to differentiate vegetations from thrombi; therefore, the results of the echocardiographic study must be interpreted with caution, taking into account the patient’s signs and symptoms and the likelihood of IE or PDA-DE.^14^

A high index of suspicion is crucial to address the right diagnosis and start the correct therapy. The mainstay of the therapy, reported in the literature for IE but also applicable to DE, is a prolonged course of intravenous antibiotics tailored on the susceptibility of the causative agent, according to the American Heart Association pediatric IE guidelines.^15^ Considering that fibrin-platelet matrix allows pathogens to survive in high concentration, the length of treatment ranges from 4 weeks in case of highly susceptible streptococcal endocarditis to 6–8 weeks in case of Gram-negative endocarditis. Overall, bactericidal antibiotics are the treatment of choice and preferred to bacteriostatic ones given the risk of treatment failure and relapses.^15^ Focusing on our case, *Enterococcus* species rarely causes endocarditis in children, with very few reports in neonates.^1,9,16,17^ Although uncomplicated enterococcal bloodstream infections usually require only single-agent antibiotic treatment, persistent bloodstream infections and endocarditis need the synergistic combination of 2 agents.^18,19^ The abovementioned guidelines recommended the combination therapy of penicillin G or ampicillin together with gentamicin for 4–6 weeks for enterococcal endocarditis caused by susceptible strains. However, the increasingly emergence of high-level vancomycin, ampicillin, and aminoglycoside resistance represents a real therapeutic challenge, precluding the use of several antibiotic combinations. In the case described, *E. faecalis* was vancomycin and ampicillin sensitive but high-level gentamicin resistant. For this reason, when simple bacteriemia was suspected, a single-agent treatment with ampicillin was started, to which cefotaxime was added when the endocarditis vegetations were suspected. In fact, the American Heart Association guidelines propose the combination of ampicillin plus ceftriaxone in aminoglycoside-resistant enterococci.^15^ Nevertheless, ceftriaxone is usually avoided in neonates considering the risk of precipitation with calcium-containing intravenous fluids and biliary sludging.^18^ Although enterococci show an intrinsic resistance to cephalosporins, the combination of an aminopenicillin and a third-generation cephalosporin has synergistic activity against these organisms, based on in vitro studies and data mainly from adults.^18^ Little evidence comes from neonatal population.^1,9,16,17^ Tam et al^18^ described a case of persistent bacteremia in a preterm infant sustained by HLAR *E. faecalis* successfully treated with ampicillin and cefotaxime. Vancomycin resistance represents another relevant issue in enterococcal endocarditis treatment. Ang et al^9^ reported a 4½-month-old extremely premature infant with endocarditis caused by *Enterococcus faecium* resistant to vancomycin, ampicillin and quinupristin-dalfopristin successfully managed with intravenous linezolid for 7 weeks. Similarly, Hapnes et al^16^ reported a case of an extremely low-birth-weight infant with a corrected gestational age of 25 weeks with persistent sepsis caused by vancomycin and high-level gentamicin-resistant *E. faecium*, complicated with an infected intraaortic thrombus. Combination therapy with linezolid and chloramphenicol (continued, respectively, for 6 and 2 weeks) allowed an excellent clinical and microbiologic response.^16^ In Table 1, we summarized all cases of neonatal enterococcal endocarditis or DE reported in literature, including ours.

**TABLE 1.** Clinical Data and Treatment of Neonates with Enterococcal Endocarditis or Ductal Endarteritis Reported in the Literature

Author	GA	Birth Weight (g)	Age at Diagnosis	Pathogens Isolated in Blood	Antibiotic Resistance	Antimicrobial Therapy	Length of Intravenous Treatment (wk)	Heparin	Site of Vegetation
Pearlman et al^1^	27	720	37 wk	*Enterococcus faecalis* (coagulase-negative staphylococcus)	NA	Vancomycin, gentamicin	4	NA	Mitral valve
Ang et al^9^	26	831	4½ mo	*Enterococcus faecium*	Vancomycin, quinupristin-dalfopristin, ampicillin, gentamicin	Linezolid	7 (plus 2 wk orally)	No	Tricuspid valve
Hapnes et al^16^	23^+^6	610	25 wk	*E. faecium*	Penicillin, ampicillin, vancomycin, teicoplanin, erythromycin, ciprofloxacin, high-level gentamicin, high-level streptomycin, quinupristin/dalfopristin	Linezolid, chloramphenicol, gentamicin	6 (linezolid), 2 (gentamicin and chloramphenicol)	No	Abdominal aorta
Parra Buitrago et al^17^	31^+^5	1010	10 d	*E. faecalis*	NA	Ampicillin, gentamicin	4–6	yes, 4–6 wk	Inferior vena cava
Present case	30	1150	1 mo	*E. faecalis*	High-level gentamicin	Cefotaxime, ampicillin	6	Yes, 4 wk	Patent ductus arteriosus

GA indicates gestational age.

The role of therapy with unfractionated or low-molecular-weight heparin has not been studied in neonates with IE and no evidence from randomized controlled trials allows to recommend or refute the use of heparin in neonates with thrombosis.^14,20^ Moreover, treatment of neonatal IE with recombinant tissue plasminogen activator has been rarely reported.^8,10,21,22^ Progressive enlargement of the vegetations despite adequate antiinfective agents, persistent positive blood cultures, severe thrombocytopenia and rapidly progressive cardiac failure were the indications for recombinant tissue plasminogen activator treatment in these reports. Although all these cases were successfully treated, the use of recombinant tissue plasminogen activator could have complications such as pulmonary, gastrointestinal and intracranial hemorrhage along with pulmonary emboli.^8,10,21,22^ To date, no specific recommendation exists about the management of anticoagulant therapy and thrombolysis in patients with IE, considering the low level of evidence available especially in neonatal population, and decisions should be made on an individual basis.^14,15^

In conclusion, IE and PDA-DE detection is rising inside neonatal intensive care units: a high index of suspicion is essential for early diagnosis and treatment, and serial echocardiograms should be performed carefully in every case of neonatal sepsis unresponsive to antibiotic therapy despite in vitro evidence of antibiotic sensitivity. Enterococcal IE and PDA-DE sustained by HLAR strains can be successfully treated with the synergistic combination of ampicillin and cefotaxime even in preterm neonates.
